# Use of the Aerosol Rabbitpox Virus Model for Evaluation of Anti-Poxvirus Agents

**DOI:** 10.3390/v2092096

**Published:** 2010-09-27

**Authors:** Chad J. Roy, Thomas G. Voss

**Affiliations:** 1 Infectious Disease Aerobiology, Microbiology Division, Tulane National Primate Research Center, 18703 Three Rivers Road, Covington, Louisiana, USA; 2 Department of Microbiology & Immunology, Tulane University School of Medicine, New Orleans, Louisiana, USA; E-Mail: tvoss@tulane.edu

**Keywords:** rabbitpox, aerosol, aerobiology, animal model

## Abstract

Smallpox is an acute disease caused by infection with variola virus that has had historic effects on the human population due to its virulence and infectivity. Because variola remains a threat to humans, the discovery and development of novel pox therapeutics and vaccines has been an area of intense focus. As variola is a uniquely human virus lacking a robust animal model, the development of rational therapeutic or vaccine approaches for variola requires the use of model systems that reflect the clinical aspects of human infection. Many laboratory animal models of poxviral disease have been developed over the years to study host response and to evaluate new therapeutics and vaccines for the treatment or prevention of human smallpox. Rabbitpox (rabbitpox virus infection in rabbits) is a severe and often lethal infection that has been identified as an ideal disease model for the study of poxviruses in a non-rodent species. The aerosol infection model (aerosolized rabbitpox infection) embodies many of the desired aspects of the disease syndrome that involves the respiratory system and thus may serve as an appropriate model for evaluation of antivirals under development for the therapeutic treatment of human smallpox. In this review we summarize the aerosol model of rabbitpox, discuss the development efforts that have thus far used this model for antiviral testing, and comment on the prospects for its use in future evaluations requiring a poxviral model with a focus on respiratory infection.

## 1. Introduction

The *Poxviridae* are members of a family of complex DNA viruses that replicate entirely in the cytoplasm of vertebrate or invertebrate cells. Two members of the family, variola virus (VARV) and molluscum contagiosum virus (MOCV) are obligate human pathogens, but others can be transmitted from animals to humans as zoonoses. VARV is the etiologic agent of human smallpox, a virus with historic impact on human health and continued potential as a biological weapon. Naturally occurring human smallpox was eradicated in 1977 after a coordinated global vaccination program. Corresponding prevention efforts, including mass vaccination campaigns of the general population were soon thereafter halted, leaving a new generation immunologically naïve and unprotected from viral infection. Nearly three decades later, serious concerns about the possibility of VARV being revived as a biological weapon were rekindled as a by-product of the worst act of bioterrorism in the history of the United States [1]. The subsequent risk assessment of approved medical products revealed how woefully unprepared we were to effectively protect and treat potential victims of a bioterrorist release involving VARV. This initiated a renewed interest in bolstering existing vaccine stocks as well as identification and evaluation of effective antivirals that demonstrate potency against poxviruses. In the absence of an animal reservoir, VARV now is maintained in two secured laboratory locations in the U.S. and Russia. Because of the increasing threat potential presented by terrorist or other organizations with intent to use biological weapons of mass destruction, development of countermeasures for VARV remain a priority for the U.S. government. The approach to VARV countermeasures includes vaccination of high risk of exposure individuals, and a post-exposure vaccine, immune globulin, or antiviral therapy for laboratory accidents or in the event of an intentionally produced exposure.

Testing and evaluation of antiviral therapeutics effective against smallpox presents a number of logistical and scientific challenges, including limited access to VARV and lack of a clinical population to better understand disease pathogenesis in the natural host (man). One of the more troublesome problems in the research and development of effective antiviral therapeutics for smallpox is the lack of a disease model that emulates the human condition and can be referenced as a benchmark standard from which other animal models are measured. Specifically, there have been few identified poxviral animal models which incorporates salient aspects of human smallpox in terms of modality of exposure, clinical development and pathogenesis of disease. Rabbitpox virus (RPXV) and specifically rabbitpox (RPX) represents one such model that shares many similarities with smallpox; the most notable characteristic being significant involvement of the respiratory system when aerosol is used as the modality of experimental infection.

## 2. Rabbitpox in Rabbits

Rabbitpox virus (RPXV), a 56 kDa double stranded DNA virus within the family *poxviridae*, is the causative agent of rabbitpox disease (RPX) in rabbits. RPX first emerged and was reported from an outbreak in rabbits in Utrecht, Netherlands [2], and later in a second outbreak at Rockefeller Institute for Medical Research in the 1930s [3,4]; the former isolate subsequently is the progenitor of the viral strain commonly used for infection studies (Table 1) and genomic sequencing [5]. In both reports of the outbreak among the rabbit colonies, it was noted that the virus quickly spread throughout the animal population, apparently through both contact housing and through aerosol transmission, producing disease in a vast majority of the animals [3]. Animals involved in these outbreaks were described as showing a range of signs indicative of a disease that is now recognized as RPX infection. Subsequent studies using RPXV in rabbits have shown that experimental viral infection can induce a similar disease observed in early reports using a variety of routes of exposure [6–11], with some routes of exposure such as intradermal (ID) inoculation being preferred by some for the study of early stage poxviral pathogenesis [12–15]. The original outbreak of RPX was described as rabbits in both a contact and noncontact housing configuration; from these descriptions, it can be presumed that RPXV was aerosol-transmitted from infected host to a naïve cohort within the colony. The fact that RPXV was an aerosol-transmitted disease in a laboratory outbreak suggests that the virus and the resulting disease (RPX) is similar to VARV in that its natural route of transmission is through the infected respiratory secretions of the selected host.

Airborne rabbit-to-rabbit RPX communicability of noncontact ID-inoculated and naïve animals has recently been described [14]. These cleverly-designed experiments demonstrate that RPX is in fact transmissible as an airborne virus when expelled via infectious aerosols or droplets by an infected host. This may be the underlying reason that early model development efforts incorporated aerosol as a preferred route of exposure for experimental infection [9–11]. The aerosol RPX model, the subject of this review, has the capacity to produce a disease that severely affects animals and when given at the appropriate dose, a lethal viral disease model.

### 2.1. Experimental Aerosol Infection

**Figure 1.**
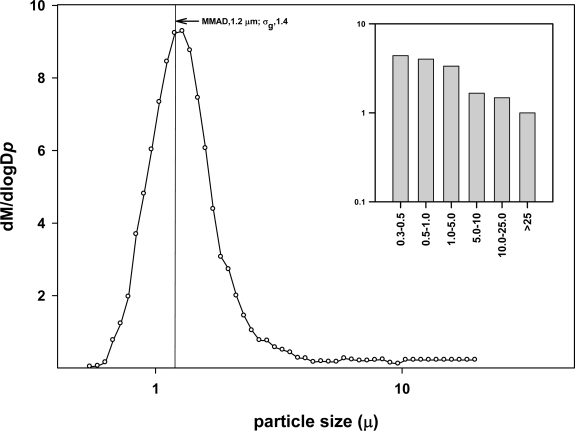
Comparative particle size distributions^1^ from aerosol generator used in experimental RPX infection and comparative particle counts of exhaled breath aerosols of a naïve NZW rabbit (inset graph). ^1^ Ordinate axis represents the mass of particles between D*_p_* and *d*logD*_p_* (D*_p_* = particle diameter). For the particle distribution generated by the nebulizer used in the experimental RPX infection, the transecting line for the distribution represents the mass median aerodynamic diameter (MMAD) and geometric standard deviation (σ_g_), respectively. The abscissa axis represents particle size in μm. The particle distributions of the animal’s exhaled breath represent cumulative particle (count) for each discrete size selective bin.

The signs and clinical development of the RPXV disease model have been detailed in a number of past reports [9,10]. The respiratory route of exposure to induce disease, or an experimental ‘aerosol’ model, was used extensively in the 1960s, and has recently been used in pathogenicity [16], antiviral [17], and vaccine efficacy studies [18]. Experimental infection with RPXV initiates with exposure to aerosols with a particle size distribution that is preferential for penetration to the tracheobronchial and pulmonary regions of the lung, with emphasis on the lower respiratory tract. Prior efforts have used particle size distributions with median diameters measuring at or under 1.0 μm[9]; recent studies also have used particle distributions similar to this size range [16–19]. It should be noted that the particle size distribution associated with normal expiration of rabbits (naïve or RPXV-infected) should be considered altogether different than the size distribution of aerosols used in experimental infection [20] (Figure 1).

Deposition studies using radiolabeled particles with a similar size distributions as used in experimental infection estimates 50–80% of an inhaled dose will penetrate to the mid to lower lung of a rabbit [21]. In contrast, the particles contained in exhaled breath of rabbits are generally more heterogeneous and will shift initial deposition of infectious aerosols in the respiratory system to the mid and upper airways (Figure 1). Experimental infection with RPXV includes artificial generation of a viral suspension using a nebulizer into either nose-only or muzzle-only inhalation exposure system. This type of inhalation exposure has been performed using unanesthetized rabbits in an unrestrained breathing configuration during acute experimental procedure. Reporting of delivered inhaled dose of RPXV can be variable based upon selected method of sampling the aerosol during experimental infection and *post hoc* analysis of the collected aerosol sample. Reproducibility of the aerosol infection may be technically difficult to transfer to another laboratory attempting to recapitulate the model because of the equipment and biocontainment facilities required to perform this type of exposure. Reported doses of inhaled RPXV in prior studies using the aerosol model to cause near complete lethality has been >50 PFU, although the variability surrounding this dose estimate is potentially large [9,17]. The variability of dosage estimates based on the type of sampling performed during exposure, inhalation aerosol system in use, and other nuances about the experimental configuration, can make it exceedingly difficult to directly compare the infectious dose across studies (Table 1).

### 2.2. Aerosol RPX Model

Following initial RPXV aerosol infection, a viral incubation lasting from two to three days ensues, similar to the timing of RPXV induced by other modalities of exposure such as intradermal (ID) inoculation [14]. High titers of RPXV developed in the respiratory system have shown to develop early (+24–36 h postexposure) in the aerosol infection model. Although temporal viral loading in the lungs of ID-innoculated animals has not been fully quanitated [14], significant respiratory involvement has been described as worsening at day 8–9 postinfection [17,22]. Notably, early viral dissemination into the ovaries and testicles has also been observed in past studies using aerosol infection [17]. The first signs of clinical disease manifesting as weight loss and hyperthermia begin to emerge on day 2–3 postexposure and continue to worsen throughout the remainder of the illness. Other noted clinical signs of RPX infection that become evident at day 2–3 postexposure include anorexia, depression, facial and cervical edema, dehydration, and diarrhea. Significant mucus membrane involvement including thick serous discharge from the eyes and nose also accompanies other clinical signs of RPX infection.

A characteristic feature of RPX disease that is similar to other poxviral infections is appearance of exthanema, and it has been the practice to shave the rabbit’s flank prior to exposure to observe development with any accuracy. The rash first appears in aerosol-infected animals as small papular lesions on day 4–5 postinfection, emerging mainly on the animal’s trunk. The rash continues to develop to vesicular lesions throughout the course of the illness, although death will often precede resolution of the rash due to the rapidly worsening clinical signs [22]. Most clinical signs associated with the aerosol model of RPX is similar to what is observed using ID inoculation model [14] with the exception of timing and development of the characteristic rash. In the ID model, the initial injection site shows infection and eventually becomes necrotic, with the appearance of secondary dermal lesions day 5–7 postexposure [14]. In the aerosol model, macular lesions are observed day 5–6 postexposure with distribution predominantly on the animal’s trunk [17].

**Figure 2.**
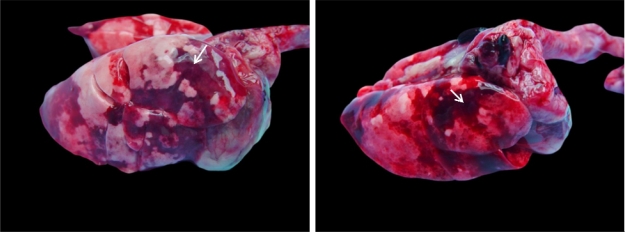
Two examples of lungs from naïve rabbits exposed to aerosolized RPXV taken at day +6 PI. The lungs are enlarged and heavy with patches of congestion, hemorrhage (arrows) and edema. Discoloration of the lung tissue defines collapsed areas of virally-induced inflammation bordered by light-colored aerated lobules.

Respiratory involvement is also pronounced in the aerosol RPX model, and the severity of effect has been noted in recent reports using this exposure modality [17]. Rabbits infected by aerosol experience an acute onset (+3 days PI) of respiratory signs including hyperventilation and dyspnea, with a rapid worsening to frank respiratory distress and open-mouth breathing by day 6–7 PI. Aerosol infected rabbits have been observed to progress quicker to profound respiratory distress than infection via ID inoculation. The rapidity of respiratory involvement in aerosol-infected rabbits is likely associated with the direct targeting of the lung during the initial exposure. The particle size distribution that has been used in experimental aerosol RPXV infection specifically impacts the lower (pulmonary) region of the lung which is considered the most susceptible region of the respiratory system. The respiratory pathology that ensues after aerosol inoculation shows dramatic lung involvement (Figure 2), with almost complete lung involvement at the time of death (Day 6–7 PI).

**Figure 3.**
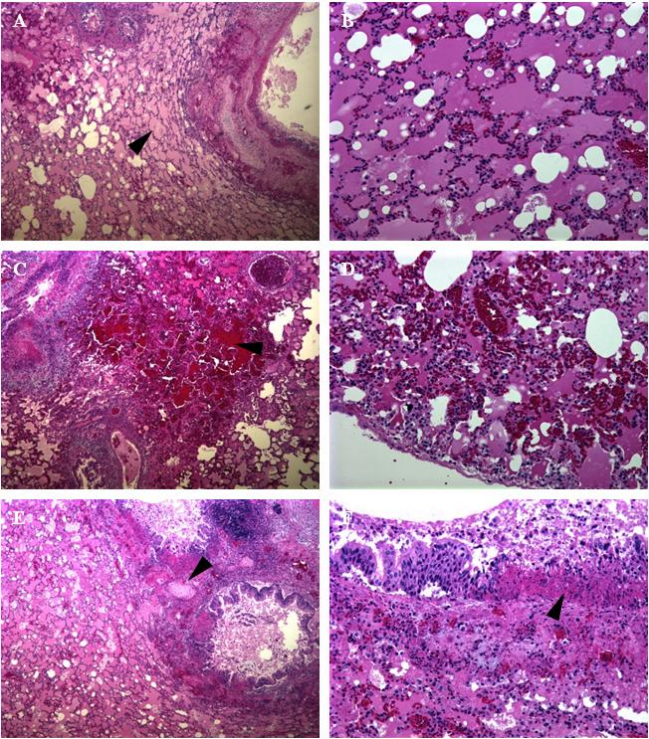
Lung pathology^1^ of aerosolized RPX in rabbits. ^1^ Lung histologic sections from naïve animals exposed to aerosolized RPXV that died on day +7 PI. **(A)** alveolar edema and necrosis of adjacent bronchiole (arrow) 50×; **(B)** diffuse alveolar edema with residual trapped air (200×); **(C)** severe hemorrhage adjacent to a necrotic airway (50×); **(D)** severe congestion of capillaries and venules with microhemorrhage (200×); **(E)** perivascular inflammation (50×); and **(F)** bronchiolar epithelial necrosis (200×).

Histologically, evidence of edema, hemorrhage, and necrosis throughout the parenchyma of the lung indicative of an acute viral infection is evident (Figure 3). The respiratory pathology associated with aerosol infection emphasizes the severity of disease in this model for testing antiviral agents.

## 3. Antivirals Used in Conjunction with Aerosol RPX Virus Infection Model

A litany of antiviral compounds have been tested using a number of the orthopox viruses and the relevant corresponding disease models which have been thoroughly reported and reviewed in the literature [23–29] and are detailed in other articles included in this special issue of *Viruses*. There are few antiviral compounds, however, that have been assessed using the aerosol RPX model, a fact that has been highlighted in comprehensive reviews of antiviral screening in a number of animal models [27]. The limited use of this particular model in the evaluation of antiviral therapeutics may have to do with practical issues rather than the biological nuances of the model itself. The RPX aerosol model uses an animal species that requires significantly more housing space than rodents and requires the use of sophisticated inhalation equipment in specialized biocontainment facilities. In addition, RPXV has also shown to be highly transmissible between infected and naïve cohorts within the same animal room [14], a phenomenon that may further confound results of antiviral evaluation studies. These factors alone may have limited the use of this model for screening large numbers of potential antiviral compounds. The aerosol RPX model also requires a low infectious dose (estimated at 1 PFU) [9] remarkably close to the estimated lethal dose (approximately 20 PFU) [17] (Table 1).

The narrow range between these two reported values (ID_50_ and LD_50_) makes it exceedingly difficult to use the model to study morbidity without causing death in RPXV-exposed animals. Increasing the dose of RPXV by aerosol (however slightly) has shown to significantly increase severity of disease and accelerate time to death, which may not be ideal for antiviral evaluation studies targeting reduction of morbidity rather than death as the primary endpoint. The low aerosol dose is in contrast to the ID inoculation model which requires between 500 and 1,000 PFU administered as a bilateral injection to ensure 100% lethality in nine week old rabbits [14].

The narrow dose range of RPXV dose established in prior studies for the ID_50_ and the LD_50_ can be potentially problematic when using aerosol as the modality of exposure because of the inability to fine tune the model to produce only disease without lethality. Aerosol exposure is inherently variable especially when using larger animals that are exposed singly, as is the case with rabbits and nonhuman primates [30]. The reasons that aerosol viral infection dose may be variable, which are not limited to RPXV exposures, is primarily due to: (1) the uncontrollability of animal respiration during exposure and (2) fluctuating viral stability when in aerosol form during the experimental infection [31]. Because limited or no control can be exerted over these factors during individual exposure, the targeted exposure dose normally fluctuates up to 15–20% between animal cohorts. The variability experienced with this modality of exposure may limit the range of preferred biological response (infection with recovery *vs.* lethality) when designing a study using the aerosol RPX model. A highly variable experimental aerosol dose may lead to an undesirable outcome by causing an overwhelming infection in some animals thereby negating or muting any potential therapeutic effects of an antiviral undergoing evaluation.

The aerosol RPX model has been used sparingly for antiviral agent evaluation. Recent efforts have included assessment of a novel antiviral and efficacy of a new generation smallpox vaccine [17–18]. Although the use of the aerosol RPX model has been somewhat limited in the last 50 years, it has shown to be a reliable and rigorous test system considering the relative variety of chemical and biologic materials that have been evaluated (Table 2). The value of the aerosol RPX model was noted in the early 1960s when it’s utility was demonstrated in a passive hyperimmune sera-based therapeutic study [7]. In this study, rabbits were experimentally infected with dry powder formulation of RPV reported as measuring approximately 1.0 μm in diameter at an estimated dose of ≈175 PFU. Thereafter, animals were administered hyperimmune sera derived and purified from convalescent rabbits from a previous study. Animals were intravenously administered with the hyperimmune sera at full strength or 1:10 or 1:100 dilutions immediately or three days postexposure. Results showed complete protection from death in the earliest treated groups treated with undiluted sera, whereas animals receiving dilutions in the early treatment group or the delayed treatment group showed signs of infection and only partial protection from death from RPX infection. This early study showed the relative severity of the model and acute action of the virus to infect and replicate in a range of tissues before a treatment can be administered.

Early studies into the antiviral potential of orally-administered thiosemicarbazone compounds (*3*-methylisothiazole-*5*-carboxaldehyde thiosemicarbazone) have also been evaluated using the aerosol RPX model, although the route of the experimental infection was not specifically noted in this work [32]. Rabbits administered either 100 or 200 mg/kg daily for four days postexposure were partially protected from lethal RPX infection, and this agent failed to fully protect in additional testing using other orthopoxviral disease models. The relatively weak antiviral potential of thiosemicarbazone compounds (isatin-β-thiosemicarbazone) was confirmed in follow-up studies using a cowpox virus (CV) model [33] and was subsequently discounted as an effective antiviral against poxviruses.

The development of (S)-1-(3-hydroxy-2-phosphonylmethoxypropyl)cytosine (cidofovir, HPMPC) as an antiviral for poxviruses has received attention in the last two decades due to its remarkable potency and relatively long intracellular half life [34]. Originally developed for use against CMV retinitis in AIDS patients and administered by intravenous infusion, cidofovir is a DNA polymerase inhibitor that can be highly specific to poxviruses [35]. Cidofovir effectively inhibits poxviruses in a number of systemic and lethal animal models and has been catalogued in reviews dedicated to the subject [25,27,35]. Cidofovir, surprisingly, has not been extensively tested using the aerosol RPX model. Recent work within our laboratories using the aerosol RPX model has shown IV-administered cidofovir to completely protect against infection and lethality when administered immediately after aerosol infection (Table 2) at a relatively low dose (10 mg/kg/day). Lower doses (1 and 0.1 mg/kg) given on the same schedule (daily for three days PI) resulted in only partial protection and development of disease. A reformulated version of cidofovir optimized for administration by inhalation is also being tested using this model [19].

A relatively newly discovered small molecule denoted ST-246, targets a major gene product that encodes a major envelope protein (p37) required for production of extracellular virus [36]. This novel mechanism of action has resulted in ST-246 showing inhibitory effects against a number of orthopoxvirus *in vitro* [36]. ST-246 is also orally bioavailable, simplifying administration compared to other drugs that require intravenous infusion such as cidofovir. The aerosol RPX model was used to test the protection afforded by administration of ST-246 in a therapeutic evaluation study. Animals were exposed to aerosolized RPXV and then received ST-246 orally at 40 mg/kg/day, for 14 days, initiating immediately, or up to four days postexposure. Results showed that groups initiating treatment immediately postexposure were completely protected from RPXV infection and death, whereas animals in the delayed administration groups (1, 2, and 3 days PI) showed signs of infection and reduced survival. Clinical signs in animals partially protected by ST-246 were remarkably similar to what has been observed in cidofovir treated rabbits exposed to aerosolized RPX. Delay of initiation of treatment with ST-246 was the most important factor in the treatment of RPX disease in this study [17]. This evaluation study highlighted the usefulness of this model system and provided essential bridging data that has since produced evaluation nonhuman primate studies with monkeypox virus and VARV [37,38] although the modality of exposure in these studies was intravenous injection.

## 4. Relevance of the RPXV Aerosol Model to the Biological Threat

Smallpox is a disease considered to be transmitted via airborne transfer of VARV in natural communicable infections; the virus has adapted to transmit between human hosts via respiratory secretions for millennia. Although eradicated through vaccination for over four decades, the threat of a bioterrorism-type attack with VARV remains. Because clinical populations are nonexistent, reliance upon poxviral disease models are a critical and scrutinized portion of screening potential therapeutics. Models that emulate the similarities in terms of what is predicted from expected modality of exposure (e.g., inhalation) are therefore increasingly relevant for evaluation of antiviral compounds. The aerosol model of RPX represents such a model system. The aerosol RPX virus model is one of the more severe poxvirus animal models available for use in the evaluation of antiviral compounds [27], although the modality of experimental infection is technically challenging, and the dose required to achieve lethality exceedingly low when compared to other experimental routes of infection. Respiratory system involvement early after experimental infection is prominent in this model; targeting of the lung can be a desirable attribute for antiviral evaluation against a disease thought to be contracted by the airborne route. The use of the RPX aerosol model in antiviral testing has been sparse thus far; this is probably due more to logistical and husbandry restraints than the desire to utilize the model [27]. The aerosol RPX model, however, should not be overlooked as an ideal intermediate test system for antiviral testing that should be considered once available rodent models have been exhausted and prior to the use of larger species for testing such as monkeypox infection in nonhuman primates.

## Figures and Tables

**Table 1.** Reported RPXV aerosol doses and survival outcome.

**PRXV/strain**	**RPXV dose (PFU/animal) (reported)**	**Group survival (%)**	**Reference**
*Utrecht/*	175	0/8 (0)	[7]
	15	1/7 (14)	
*Utrecht/*Rockefeller	50	1/8 (12)	[9]
	250	1/9 (11)	
*Utrecht*	2,860 (1,140–5,000)	0/6 (0)	[19]
*Utrecht*	296 (96–468)	0/6 (0)	[19]

**Table 2.** Compounds and biologics evaluated using lethal aerosol RPX infection model.

**RPXV dose (reported) strain/(PFU/animal) (dose range)**	**Test compound**	**Effective treatment regimen**		**Reference**
		**Complete protection from lethality and infection**	**Partial protection from death**	
*Utrecht*/175 (146–175)	purified hyperimmune sera	10 mL; 1:10, 1:100 dilution/1day PI or 10 mL/3 days after PI	10 mL/1:10 dilution/3 days PI	[7]
*Utrecht/–*	thiosemicarbazone (M&B 7714)	–	100 or 200 mg/kg daily for 4 days	[32]
*Utrecht*/2,860 (1,140–5,000)	ST-246 (tecovirimat)	40 mg/kg daily for 14 days initiating immediately (0 h PI)	40 mg/kg daily for 14 days initiating +24, +48 or +72 h PI	[19]
*Utrecht*/296 (96–468)	cidofovir (vistide)	10 mg/kg daily for 3 days initiating either immediately or +24 h PI	1 mg/kg daily for 3 days initiating either immediately or +24 h PI	[17]
